# Sterol analysis in cancer cells using atmospheric pressure ionization techniques

**DOI:** 10.1007/s00216-025-06126-1

**Published:** 2025-10-02

**Authors:** Pia Wittenhofer, Juan F. Ayala-Cabrera, Laila Orell, Florian Uteschil, Sven W. Meckelmann, Oliver J. Schmitz

**Affiliations:** 1 Applied Analytical Chemistry, University of Duisburg-Essen, Universitaetsstrasse 5, 45141 Essen, Germany; 2 Department of Analytical Chemistry, University of the Basque Country (UPV/EHU), Sarriena Auzoa, 48940 Leioa, Spain

**Keywords:** Heart-cutting 2D-LC, Liquid chromatography ion sources, Ion suppression, Tube plasma ionization, Atmospheric pressure ionization

## Abstract

The analysis of sterol biosynthesis poses analytical challenges such as vast concentration differences between precursor molecules and cholesterol as well as the substantial interferences between the sterols and the matrix, resulting in strong ion suppression. LC-MS and 2D-LC-MS may address some of these challenges, but for sensitive and robust detection, ionization plays a key role. Electrospray ionization (ESI) stands out for its capability to generate ions from polar and nonpolar compounds, rendering it suitable for a broad spectrum of analytes, including sterols. Atmospheric pressure chemical ionization (APCI) demonstrates efficacy for compounds exhibiting moderate to low polarity, thereby facilitating efficient ionization of sterols. Tube plasma ionization (TPI) is a relatively new technique that promises sensitive ionization across a wide polarity range, indicating its potential utility for sterol analysis. Here, a new tube plasma ion source for LC-MS was developed and compared with established ion sources (ESI and APCI) for sterol analysis. The TPI source showed comparable LOQs to APCI and clearly outperformed ESI in terms of sensitivity. Both TPI and APCI provided stable signals during extended measurement series, while ESI suffered from pronounced ion suppression. Application to human plasma, HepG2 cells, and liver tissue demonstrated that TPI provided results in close agreement with APCI, highlighting its robustness for sterol quantification in complex biological matrices.

**Graphical abstract:**

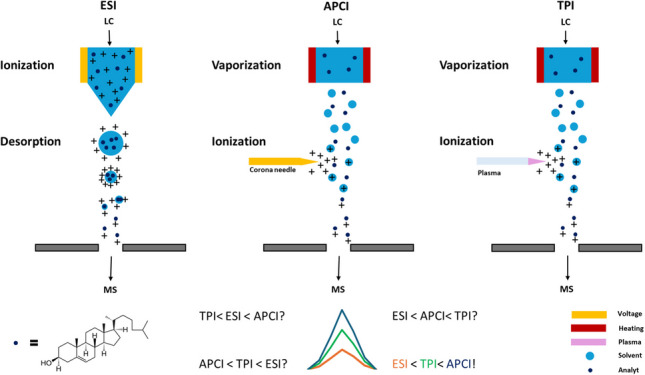

**Supplementary Information:**

The online version contains supplementary material available at 10.1007/s00216-025-06126-1.

## Introduction

Mass spectrometry (MS) has evolved into a versatile analytical technique with a wide range of applications. When selecting the most suitable MS-based methodology, the ion source is one of the key elements that might be wisely selected depending on the target compounds to be analyzed. Among the variety of ionization techniques available, electrospray ionization (ESI) and atmospheric pressure chemical ionization (APCI) stand out as the most used atmospheric pressure ionization (API) sources, offering a soft and efficient ionization for analytes of different polarities [1–3].

ESI represents the most employed ionization technique in liquid chromatography–mass spectrometry (LC-MS) due to its easy operation and the possibility to efficiently ionize a wide range of compounds [4–7]. Thus, ESI has found extensive applications in the analysis of compounds presenting an acidic or basic functional group with a huge range of polarities, including peptides, proteins, nucleic acids, pesticides, or lipids, among others [8–13]. In contrast, APCI is particularly suitable for compounds with moderate to low polarity since the ionization, utilizing corona discharges, happens by ion–molecule reactions in the gas phase [7, 14]. Accordingly, APCI has demonstrated an efficient ionization for the analysis of pharmaceuticals, pesticides, sterols, lipids, environmental pollutants, and natural products [13, 15–20], among others.

Although ESI and APCI have been extensively used for MS-based applications, in recent years, the interest in other API techniques is rising with the idea of extending the chemical space of analytical determinations [3]. In this sense, atmospheric pressure plasma-based ion sources have emerged as an alternative to overcome the typical limitations of ESI or APCI such as high ion suppression or low ionization efficiency of polar compounds, respectively [21]. Plasma-based ionization has been applied to a wide range of applications such as the analysis of environmental toxins and contaminants, explosives and chemical warfare agents, food and drinks, drugs, lipids, alkyl alkanolamine, methyl palmitate, soy oil glycerol, and potential biomarkers for cancer [22–28]. Among the plasma sources, those based on dielectric barrier discharges (DBD) seem to be one of the most suitable approaches for analytical methodologies [29]. These ionization techniques usually consist of two electrodes, which can be arranged at different positions. The electrodes are typically separated by a dielectric barrier or insulator with a gas passing through [21, 29–31]. By applying a sinusoidal voltage [32–34] or high-voltage pulses [35–37], the DBD is formed, leading to the formation of a non-equilibrium plasma at room temperature. In the ignition stage, electrons collide with the molecules in the supplied gas (usually helium or argon) causing a chain reaction that ignites the plasma [29]. The plasma can be controlled by changing the voltage, frequency, pulse width, and gas flow, offering parameters to tailor the ionization to the molecules of interest. This plasma has a temperature of around 30 °C, being even suitable for the detection of volatiles or thermally labile molecules [38]. Typically, this soft ionization technique leads to the formation of [M]^+•^ or [M + H]^+^ ions. Plasma-based ion sources have been used for GC-MS, LC-MS [36, 39–41], or ambient ionization mass spectrometry (AIMS) [22, 24–26, 42, 43]. Recently, a specific setup of plasma ion sources, so-called tube plasma ionization sources (TPI), was developed for GC-MS applications. In this configuration, the high voltage is applied to a pin electrode arranged inside the dielectric tube, whereas there is no ground electrode. This technique offered advantages in terms of simplicity, sensitivity, and versatility, making it a promising option for the analysis of a wide polarity range of compounds [27, 44].

Here, we describe, for the first time, a TPI ion source that enables the coupling of LC with mass spectrometry for the analysis of sterols. These lipids are essential for cellular functioning and are discussed as possible targets in cancer therapy [45–48]. However, their analysis is challenging because of the structural similarity of the sterols involved in the biosynthesis as well as the vast concentration differences within a sample ranging from nM to mM. To develop effective treatment strategies, the individual sterols of the biosynthetic pathway have to be quantified accurately. We investigated the performance of the lab-made TPI as well as commercial ESI and APCI ion sources, as different plasma-based ion sources have already shown comparable or even better results than ESI and APCI [22, 27] and demonstrate lower matrix effects than ESI [49]. For the comparison, the characterization was performed according to the International Council for Harmonisation (ICH) guidelines [50]. In addition, the ion suppressions and signal stability are thoroughly examined, and all methods were applied to different matrices to demonstrate their application in a wide field.

## Materials and methods

### Chemicals and reagents

2,3-Oxidosqualene (92%), cholesta-5,7-dien-3β-ol (7-dehydrocholesterol) (> 99%), cholest-5-en-3β-ol (cholesterol) (> 99%), cholesterol ^2^H_7_ (> 99%), cholest-7-en-3β-ol (lathosterol) (> 99%), lathosterol ^2^H_7_ (> 99%), lanosterol (> 99%), lanosterol ^2^H_6_ (> 99%), cholesta-5,24-dien-3β-ol (desmosterol) (> 99%), desmosterol ^2^H_6_ (> 99%), 4,4-dimethycholesta-8(9),24-dien-3ß-ol (T-MAS) (> 99%), T-MAS ^2^H_6_ (> 99%), cholesta-8(9),24-dien-3ß-ol (zymosterol) (> 99%), zymosterol ^2^H_5_ (> 99%), dihydrolanosterol (> 99%), dihydrolanosterol ^2^H_7_ (> 99%), cholesta-5,7,24-trien-3ß-ol (dehydrodesmosterol) (> 99%), cholesta-7,24-dien-3ß-ol (dehydrolathosterol) (> 99%), 4,4-dimethycholesta-8(9),14-dien-3ß-ol (dihydro FF-MAS) (99%), 4,4-dimethylcholesta-8(9)-en-3ß-ol (dihydro T-MAS) (99%), 4,4-dimethycholesta-8(9),14,24-trien-3ß-ol (FF-MAS) (> 99%), squalene (≥ 98%), and cholesta-8(9)-en-3ß-ol (zymostenol) (> 99%) were purchased from Avanti Polar Lipids (Alabaster, USA). Acetic acid, n-hexane, ammonium formate (all LC-MS grade), and potassium hydroxide (> 85%) were purchased from Merck (Darmstadt, Germany). Methanol and 2-propanol (both LC-MS grade) were purchased from Avantor (Darmstadt, Germany). LC-MS grade formic acid was from Thermo Fisher Scientific (Schwerte, Germany). Human plasma was purchased by Sigma-Aldrich (Traufkirchen, Germany). HepG2 cells were purchased by CLS Cell Lines Service GmbH (Eppelheim, Germany) and cultivated as described by Tötsch et al. [9]. All equipment and materials for cell cultivation were purchased from Greiner Bio-One (Kremsmünster, Austria). Desalted and filtered ultrapure water with a resistivity of 18.2 M Ω/cm was generated by a water purification system (Sartorius, Goettingen, Germany).

### LC-MS analysis

The chromatographic methods were previously described by Wittenhofer et al. [51]. Briefly, chromatographic separation was performed using a 1290 Infinity LC system coupled to an Agilent 6470 LC/TQ instrument. The Agilent Infinity 1290 heart-cut LC system was equipped with two 1290 Infinity pumps, a 1290 Infinity autosampler, a 1290 Infinity column oven, and two 1290 Infinity valves (a 6-port/2-position and an 8-port/2-position valve) (Agilent Technologies, Waldbronn, Germany).

A Kinetex PFP column (1.7 µm, 2.1 × 30 mm) (Phenomenex, Aschaffenburg, Germany) was used as the first dimension, a Nucleodur Sphinx (30 × 4 mm, 1.8 µm) (Macherey–Nagel, Düren, Germany) as trap column, and an InfinityLab Poroshell 120 EC-C18 column (1.7 µm, 2.1 × 100 mm) (Agilent, Waldbronn, Germany) as the second dimension. In both dimensions, a binary gradient consisting of eluents A (water) and B (methanol/water (99/1; v/v) both with 5 mmol/L ammonium formate and 0.1% formic acid was used. The gradient of the first dimension was 0 min, 40% B; 1 min, 40% B; 1.01 min, 75% B; 15 min, 80% B; 15.01 min, 100% B; 18 min, 100%; 18.01 min, 40% B; and 22 min, 40% B and for the second dimension was 0 min, 40% B; 10.5 min, 40% B; 10.51 min, 79% B; 18.5 min, 89% B; 18.51 min, 100% B; 22 min, 100% B; 22.01 min, 40% B; and 23.5 min, 40% B. The columns were maintained at 45 °C. The flow rate was 0.3 mL/min in the first dimension and 0.6 mL/min in the second dimension. The heart cut was performed from 7.0 to 9.5 min, and the injection volume was 5 µL. For ion source development and initial optimization, 1D LC-MS (only first dimension without heart cut) was primarily used due to the shorter analysis time. For complex biological samples, however, 2D-LC was employed to ensure sufficient separation and reduction of matrix effects for samples with high cholesterol content. This entire setup was also validated under these conditions.

Ionization was carried out in the positive mode using an AJS source, an APCI, and an in-house built TPI ion source. The Agilent Jet Stream ion source (AJS) from Agilent Technologies (Santa Clara, USA) was used with the following parameters: gas temperature 170 °C, drying gas flow 13 L/min, nebulizer gas pressure 45 psi, sheath gas temperature 150 °C, sheath gas flow 3 L/min, capillary voltage 6000 V, nozzle voltage 1600 V, and fragmentor voltage 120 V. The APCI source from Agilent Technologies (Santa Clara, USA) was operated with the following parameters: gas temperature 350 °C, drying gas flow 3 L/min, vaporizer temperature 475 °C, vaporizer flow pressure 3 psi, capillary voltage 2700 V, corona current 8 µA, and fragmentor voltage 80 V. The home-built TPI source was operated with the following parameters: gas temperature 325 °C, drying gas flow 3 L/min, vaporizer temperature 425 °C, vaporizer flow pressure 15 psi, capillary voltage 900 V, fragmentor voltage 120 V, plasma frequency 12.5 kHz, pulse width 2 µs, plasma voltage of 2 kV, and gas flow (argon) 325 mL/min. Each ion source was optimized to the most sensitive conditions for sterols using the most intense ion as precursor (typically [M + H–H_2_O]^+^). Detection was carried out in scheduled multiple reaction monitoring (sMRM) using the optimized parameters for each transition shown in Table S1, and the cycle time was 500 ms.

### Comparison of different ion sources

Each ion source was characterized by validation according to the ICH Guideline [50] and the resulting figures of merit were used for comparison. Therefore, concentration series ranging from 0.01  to 90,000 nmol/L of all mentioned sterols were analyzed (*n* = 3). Only for cholesterol, a second validation with concentration series ranging from 100  to 9,000 µmol/L was done for sample quantification (*n* = 3). Each of the standards was spiked with 10,000 nmol/L of deuterated sterols (only for cholesterol ^2^H_7_, a concentration of 100,000 nmol/L was used) as internal standards. Moreover, human plasma, HepG2 cells, and beef liver tissue samples (from a supermarket in Essen, Germany) were analyzed with each ion source to compare their performance under matrix load. The samples were prepared by a liquid–liquid extraction using water/n-hexane. The extraction was performed with hydrolysis of esterified sterols to obtain the extract with esterified and free sterols. Details of the extraction are provided in the supplementary material (p.2).

### Ion source development

**Fig. 1:**
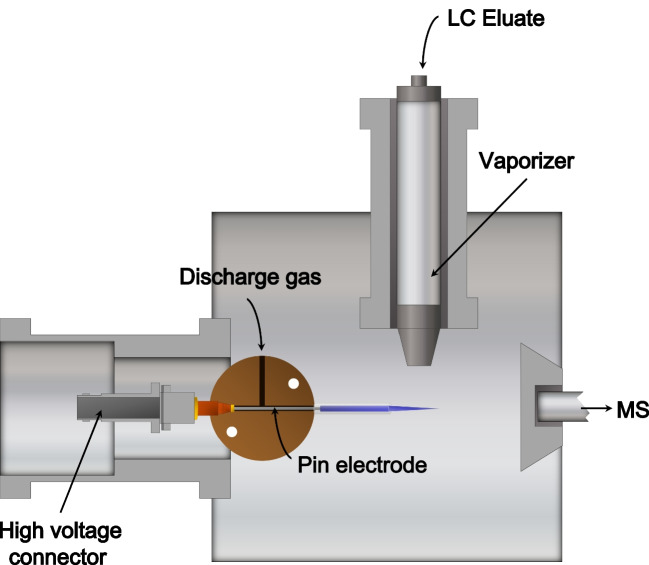
Schematic setup of the LC-TPI ionization source. This source setup offers a controlled atmosphere for the ionization region, which helps to (i) increase repeatability, (ii) promote specific reagent-assisted ionization, and (iii) reduce the matrix effect as well as MS maintenance

To adapt the TPI probe for LC-MS configurations, an aluminum housing similar to the existing LC-APCI housing from Agilent Technologies was machined. Thus, the TPI probe is positioned in a depth-adjustable cylinder which is located in front of the mass spectrometer inlet. Orthogonally, a nebulizer is inserted to facilitate the evaporation of the LC eluate before the ionization as shown in Fig. 1. The home-built TPI probe consists of a tapered 0.4 mm stainless-steel needle located inside a quartz tube (I.D. 0.6 mm) and is mounted on-axis to the mass spectrometer inlet in comparable spatial arrangement to the corona discharge needle of the APCI source. The stainless-steel needle is attached to the high voltage through a connector which is positioned inside a T-piece from polyamide which interconnects the needle and the high voltage while the discharge gas is orthogonally introduced into the T-piece.

## Results and discussion

### Ionization behavior and efficiency by API sources

**Fig. 2:**
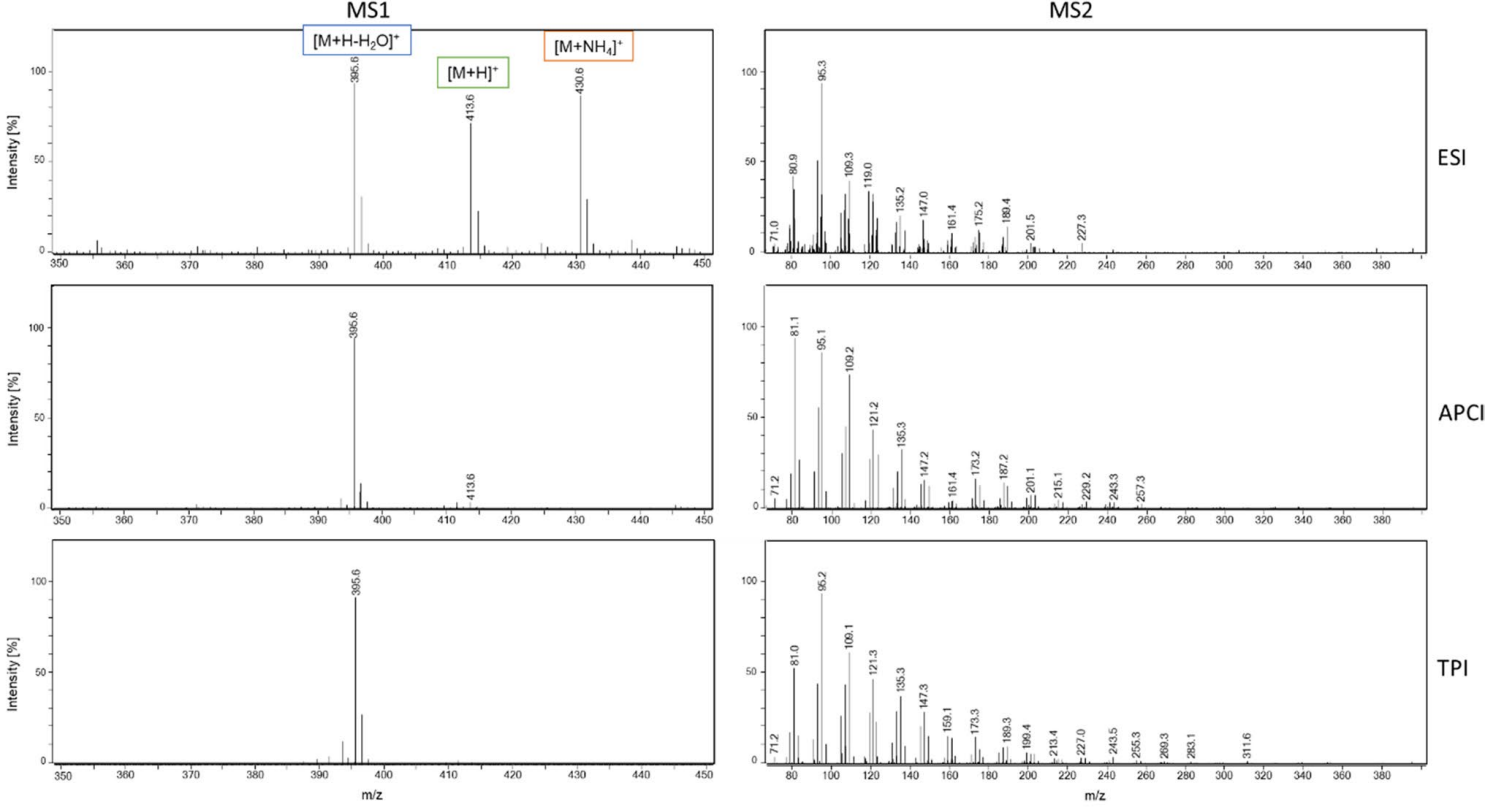
Adduct formation (full MS left) and fragmentation patterns (MS/MS right) of T-MAS with ESI, APCI, and TPI in positive ion mode

Typically, cholesterol and sterols, which have a similar structure, lose water during ionization and [M + H-H_2_O]^+^ ions are formed. To make a comparison of the ion sources, the ionization and fragmentation behavior must therefore be considered beforehand. As can be seen in Fig. 2 for the analysis of T-MAS with ESI (cholesterol and FF-MAS are shown in Figures S1 and S2), several adduct ions are formed, including [M + H] +, [M + NH_4_] +, and [M + H–H_2_O]^+^ (promoted by the additives in the mobile phase), each with comparable intensity. By contrast, APCI and TPI predominantly generate the [M + H–H_2_O]^+^ ion. This distribution of the signal across multiple adducts in ESI means that, for quantitative analysis relying on a single SRM transition, the effective sensitivity can appear lower. It should be noted, however, that this does not represent a general limitation of ESI, but rather a methodological consequence of transition selection for targeted quantification. When collision energy is applied, the *m*/*z* 95 is often obtained (Table S1), which results from the fragmentation of the carbon chain of the sterols. When comparing the MS/MS spectra, the same fragmentation patterns are obtained as expected (Fig. 2).

**Fig. 3:**
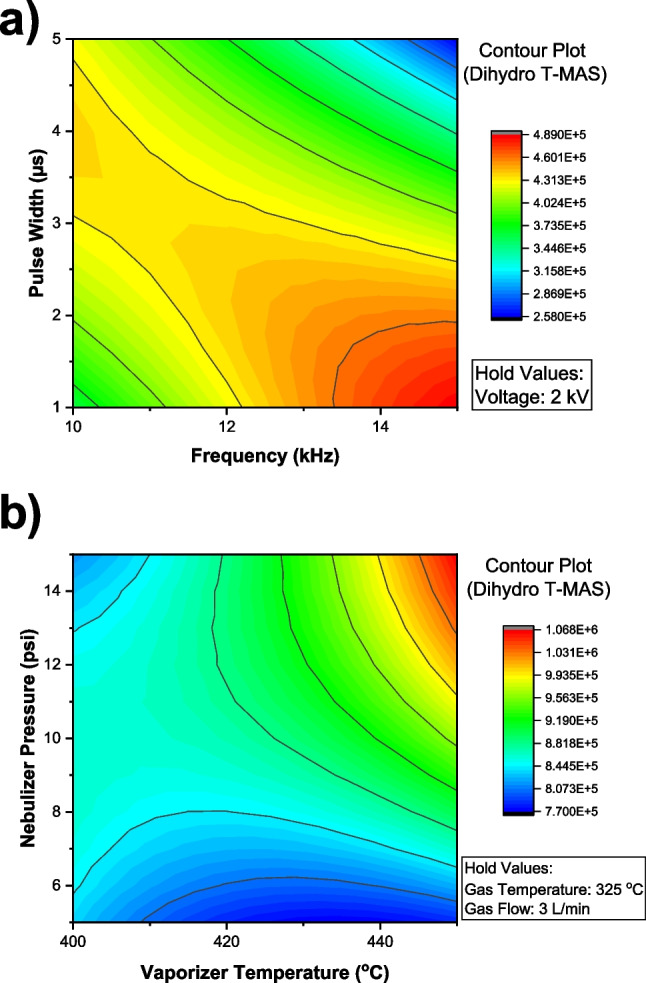
Contour plots of ionization parameters of the TPI source for **a** dihydro T-MAS and **b** cholesterol

After that, source and plasma-related parameters for TPI were carefully optimized to increase the ionization efficiency of the analytes employing designs of experiments (ESI and APCI parameters are based on those of Wittenhofer et al. [51]). Regarding plasma-related parameters, a Box-Behnken design with one block and three centered points was carried out, and the limits of the model were established at 10–15 kHz for the frequency, 1–5 µs for the pulse width, and 2–2.4 kV for the voltage. In general, it was observed that for most sterols, the interaction between plasma frequency and pulse width was the most statistically significant factor influencing ionization efficiency. However, this was not the case for cholesterol, which exhibited very high and stable signal intensity across all tested settings. Instead, dihydro T-MAS was a more representative analyte due to its moderate abundance and pronounced sensitivity to variations in plasma conditions. As shown in the contour plot for dihydro T-MAS (Fig. 3a and b), there is an inverse correlation between frequency and pulse width, and higher frequencies require shorter pulse widths to achieve optimal ionization. Based on these interactions, and considering a compromise across analytes, the plasma parameters were set to a frequency of 12.5 kHz, a pulse width of 2 µs, and a voltage of 2 kV.

Concerning source parameters, another Box-Behnken design with three blocks and three center points per block was used with the limits set as follows after pre-optimization: (i) gas temperature (300–350 ºC), (ii) vaporizer temperature (400–450 ºC), (iii) gas flow (2–4 L min^−1^), and (iv) nebulizer flow pressure (5–15 psi). In general, it could be observed that in general high temperatures and flow led to a more efficient ionization, as shown in Fig. 3. However, the trends were compound dependent, requiring achieve a compromise situation (Figure S3). Thus, the temperature was set at 425 °C and 325 °C for the vaporizer and gas temperature, while the vaporizer flow pressure and gas flow were fixed at 15 psi and 3 L min^−1^, respectively.

### Signal stability

**Fig. 4:**
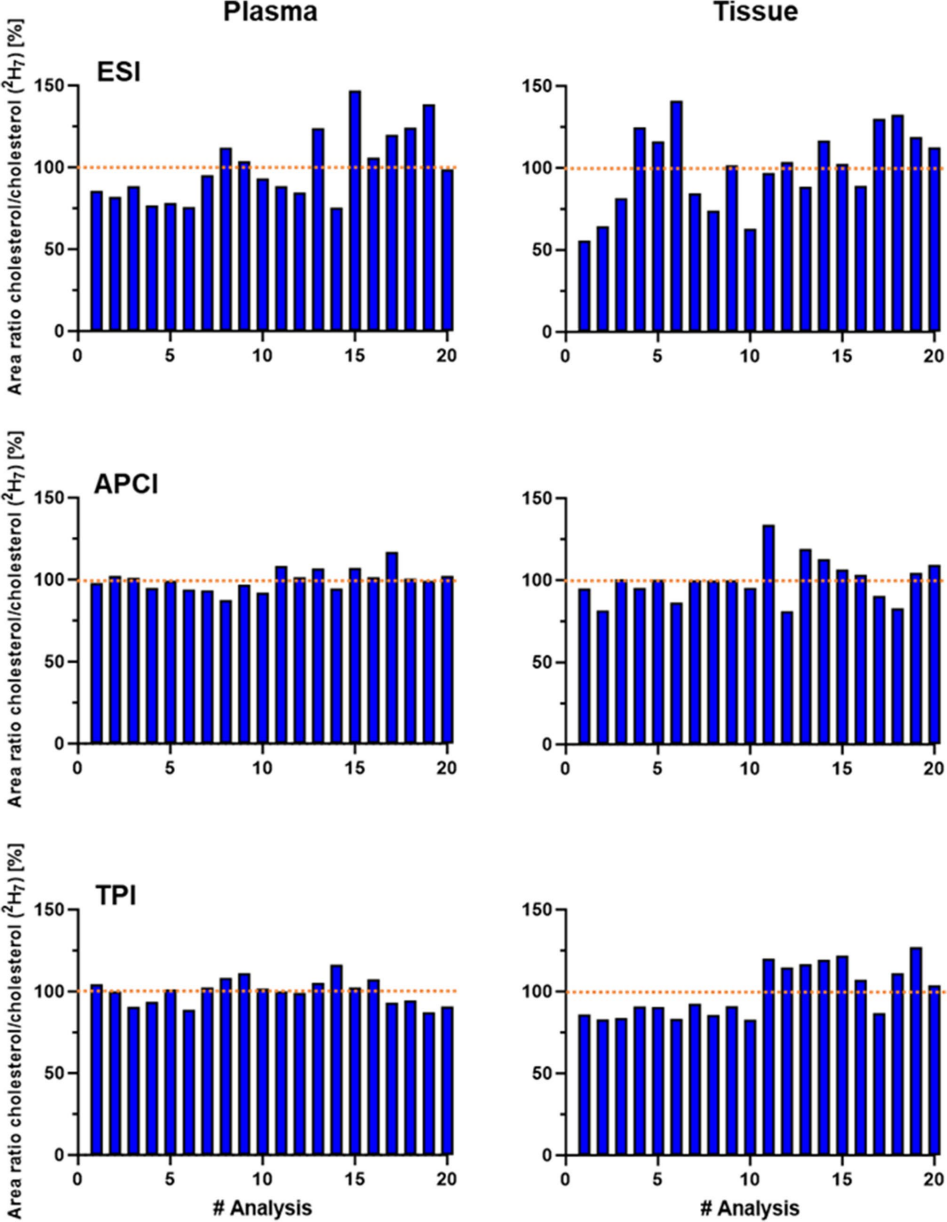
Signal stability of the ESI, APCI, and TPI ion sources evaluated using human plasma (left) and liver tissue (right) extracts over 26 h and a total of 40 consecutive injections. The area ratio of cholesterol to deuterated cholesterol was used as the stability indicator and normalized to the mean value

Delivering consistent results over long measurement series is essential. Depending on the sample and sample quantity, duration of the analysis, solvent, and additions to the mobile phase, there is a slow to rapid deterioration of the ionization efficiency in the ion source. Thereby, the observed signal or peak area must remain stable over a long period. For this purpose, plasma and tissue samples were injected alternately for 26 h, resulting in a total of 40 sample injections (20 injections each). Figure 4 shows the relation of the peak areas between cholesterol and the corresponding internal standard cholesterol ^2^H_7_ of the plasma measurements (left) and the tissue measurements (right). Figure S4 shows the peak areas of the cholesterol alone.

The APCI and TPI show stable signals with only a few fluctuations compared to ESI. A little stronger increases in the area ratio can be observed for the TPI source when analyzing tissue samples. Here, an increase can be observed which is related to a new injection vial that was used after 10 injections to avoid evaporation effects of the sample. ESI is known for its strong matrix interferences and has shown rather inconsistent results (Fig. 4). This leads to a need for a much larger set of internal standards—preferably one for each compound—when ESI is used for quantification.

### Ion suppression

**Fig. 5:**
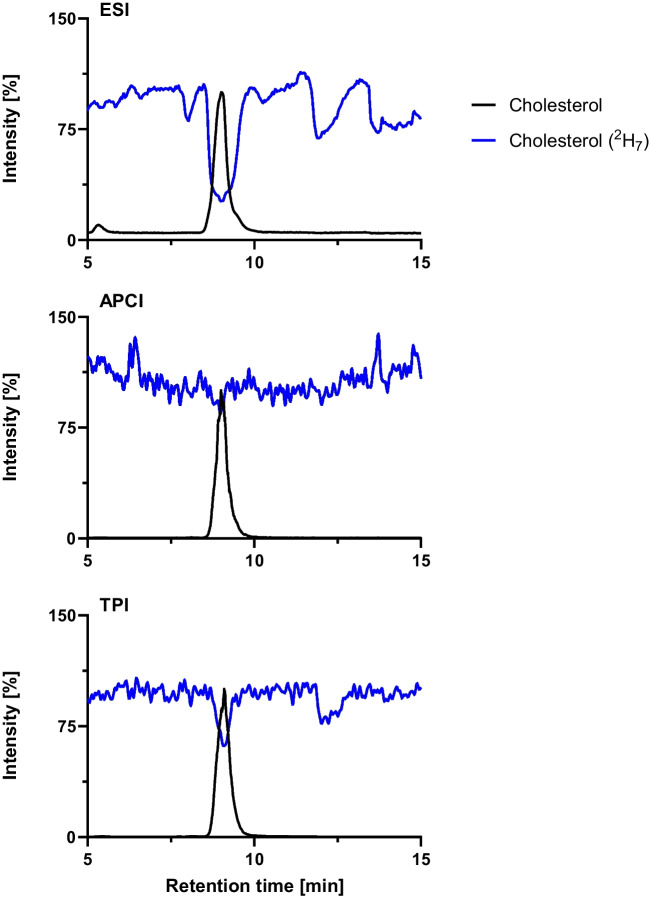
Comparison of the ion suppression strength in the detection of high matrix and analyte (cholesterol, black) concentrations compared to the signal of the internal standard (cholesterol ^2^H_7_, blue), which is added after separation via a T-piece with ESI, APCI, and TPI

ESI is known to be prone to ion suppression at high concentrations of analytes and/or matrix [52, 53]. This effect was also evaluated to assess the matrix impact using the different API source by injecting a plasma sample and adding the internal standard cholesterol ^2^H_7_ via a T-piece after the separation column. The change in the intensity of the signal of the cholesterol ^2^H_7_ during the analysis is highlighted in Fig. 5 together with the measured native cholesterol. As can be seen, both TPI and APCI exhibit stable signals throughout the analysis, in clear contrast to ESI, which suffers from pronounced ion suppression at several retention windows. In fact, APCI shows the most stable performance with minimal suppression, underscoring its robustness for sterol analysis. TPI likewise demonstrated strong resistance to ion suppression, making it a promising alternative. Overall, both techniques clearly outperform ESI under these conditions. Stronger ion suppression was observed during the elution of cholesterol itself as the concentration in a common plasma sample is at 4.14 µM [54].

### Characterization according to ICH guidelines

The method characterization for low and high cholesterol content in standards and samples followed the guidelines of the ICH [50]. A signal-to-noise ratio of three was applied for determining the limit of detection (LOD), and nine for the limit of quantification (LOQ). Furthermore, a carry-over of no more than 20% from higher concentrations was established as a compliant threshold. All LODs and LOQs were determined using calibration curves prepared in solvent. Based on these criteria, the upper limit of quantification (upper LOQ) and the coefficient of determination *R*^2^ of the linear fit were established. Table 1 presents the validation results for the heart-cut 2D-LC-MS method for each analyte and ion source, including the internal standard utilized for instrument variation calculation, LOD, LOQ, upper LOQ, slope, and regression coefficients. In Table S2, the results of the LC-QqQ-MS method (one dimension) for lower cholesterol concentrations are shown.

**Table 1:** Method characterization (heart-cut 2D-LC-QqQ-MS) according to ICH Guideline [50]

Analyte	Internal standard	LOD [nmol/L]^a^			Linear range [nmol/L]						Slope			*R*^2c^		
					LOQ^b^			Upper LOQ^b^								
		ESI	APCI	TPI	ESI	APCI	TPI	ESI	APCI	TPI	ESI	APCI	TPI	ESI	APCI	TPI
2,3-Oxidosqualene	Lanosterol (^2^H_6_)	1000	1000	1000	1000	1000	1000	30,000	90,000	90,000	2.612	3.621	3.067	0.966	0.874	0.945
7-Dehydrocholesterol	Desmosterol (^2^H_6_)	1000	10	100	1000	30	100	10,000	90,000	30,000	2.679	1.852	2.204	0.879	0.944	0.945
Cholesterol	Cholesterol (^2^H_7_)	1000	10	1000	1000	30	1000	9,000,000	9,000,000	9,000,000	8.927	10.395	10.051	0.999	0.999	0.999
Dehydrodesmosterol	Desmosterol (^2^H_6_)	300	30	30	1000	30	30	60,000	60,000	90,000	2.051	1.954	2.881	0.899	0.991	0.992
Dehydrolathosterol	Zymosterol (^2^H_5_)	1000	10	30	1000	30	30	10,000	60,000	60,000	2.262	2.387	2.219	0.9	0.962	0.962
Desmosterol	Desmosterol (^2^H_6_)	1000	3	10	1000	10	30	90,000	60,000	90,000	1.726	2.24	1.994	0.961	0.964	0.941
Dihydro-FF-MAS	Lanosterol (^2^H_6_)	300	10	30	300	30	30	30,000	90,000	90,000	2.468	2.255	2.029	0.932	0.964	0.968
Dihydrolanosterol	Dihydrolanosterol (^2^H_7_)	1000	3	30	1000	10	30	90,000	90,000	90,000	0.926	0.994	0.986	0.977	0.976	0.968
Dihydro-T-MAS	Dihydrolanosterol (^2^H_7_)	300	3	10	300	10	30	10,000	60,000	90,000	7.496	2.026	1.638	0.981	0.974	0.971
FF-MAS	Lanosterol (^2^H_6_)	1000	10	30	1000	30	30	10,000	60,000	90,000	3.269	1.335	2.072	0.931	0.935	0.935
Lanosterol	Lanosterol (^2^H_6_)	1000	3	10	1000	10	30	60,000	60,000	90,000	0.995	1.54	1.822	0.945	0.959	0.949
Lathosterol	Lathosterol (^2^H_7_)	1000	100	100	1000	100	100	10,000	60,000	60,000	1.648	1.243	1.214	0.924	0.965	0.943
Squalene	Lanosterol (^2^H_6_)	10,000	1000	1000	10,000	1000	1000	90,000	90,000	90,000	0.158	0.346	0.298	0.848	0.971	0.973
T-MAS	T-MAS (^2^H_6_)	1000	30	30	1000	30	30	90,000	90,000	90,000	1.962	2.284	2.386	0.978	0.964	0.96
Zymostenol	Zymosterol (^2^H_5_)	1000	30	30	1000	10	100	30,000	90,000	90,000	3.111	4.563	3.838	0.949	0.946	0.945
Zymosterol	Zymosterol (^2^H_5_)	1000	3	10	1000	10	10	30,000	90,000	90,000	2.612	3.621	3.067	0.968	0.996	0.979
Median value		1000	10	30	1000	30	30	30,000	90,000	90,000	3,294	4.25	2.892	0.947	0.964	0.961

*At LOQ; ^a^S/N ≥ 3; ^b^S/N ≥ 9 and accuracy ± 20%; ^c^*R*^2^ of a 1/x^2^-weighted calibration curve

As in the previous experiments, the characterization of the method shows lower values for ESI than for APCI and TPI. When comparing the APCI and TPI, for around half of the substances, the APCI is more sensitive in terms of lower LODs and outperforms the TPI. The LOQ and upper LOQ values are on average the same for both sources and are therefore comparable. This trend is further illustrated by the box plots of LOQ values in Figure S5.

When using the ESI ion source, a notably smaller linear range is observed. This is primarily due to strong ion suppression, which significantly reduces the intensity of the internal standard or even causes it to become undetectable. This issue is less pronounced or absent when using TPI and APCI. Skubic et al. [54] reported LODs between 60 and 150 nmol/L for most cholesterol biosynthesis intermediates, which are slightly higher than the values obtained in this study using APCI (median: 10 nmol/L) and TPI (median: 30 nmol/L). The median lower LOQ for both APCI and TPI was 30 nmol/L, also lower than the values reported by Skubic et al. Exceptions were observed for squalene and 2,3-oxidosqualene, where LOQs of 1000 nmol/L were determined for both ion sources. For APCI and TPI, upper LOQs ranged from approximately 60,000 to 90,000 nmol/L. By applying alternative transition settings for cholesterol, an upper LOQ of 9 mmol/L was achieved, enabling the quantification of samples with very high cholesterol concentrations. However, this is only feasible when separation is performed via heart-cutting. If transition settings are modified in the one-dimensional method, the signal for coeluting isomers sharing the same m/z as cholesterol (e.g., lathosterol) is lost. In contrast, ESI yielded much higher LOD, lower, and upper LOQ values, likely due to lower ionization efficiency and pronounced ion suppression, which ultimately makes reliable quantification in complex samples impractical.

### Applications

**Fig. 6:**
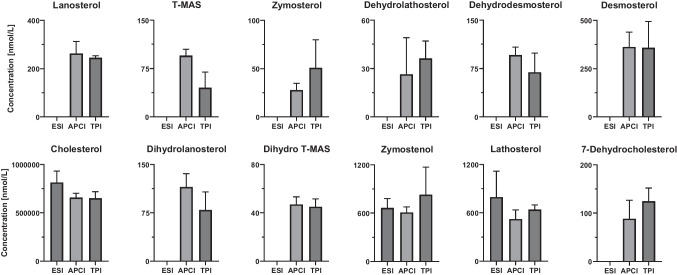
Analysis of extracted HepG2 cells. The same samples were analyzed and quantified using LC-LC with ESI, APCI, and TPI (*n* = 3)

Human plasma, HepG2 cells, and liver tissue were extracted to evaluate which ion source is best suited for analyzing compounds from sterol biosynthesis. Prior to extraction, all samples were hydrolyzed to convert esterified sterols to their free forms (see Supplementary Material p. 2). The concentrations of cholesterol biosynthesis intermediates in HepG2 cells are presented in Fig. 6, while the corresponding data for human plasma and liver tissue are provided in the Supplementary Material (Figures S5 and S6). Due to the high detection limits of ESI, many sterols could only be quantified using APCI and TPI. Both ion sources yielded very similar concentration values for most analytes (Fig. 6). This can be clearly seen for the more abundant sterols in HepG2 cells, such as lanosterol, desmosterol, cholesterol, and lathosterol. The cholesterol result obtained via ESI was slightly higher than those from APCI and TPI, likely due to increased interferences affecting the internal standard response. The observed standard deviations are small considering that the entire workflow includes cell counting, extraction, and analysis (Fig. 6). Comparable results were also obtained when analyzing human plasma (Figure S6, lower standard deviation). Only in the liver samples (Figure S7) are the differences between the APCI and TPI ion sources more pronounced. This may be due to the homogeneity of the tissue sample. Importantly, in real biological samples, certain intermediates such as zymosterol or dehydrolathosterol could only be reliably detected using APCI and TPI, while they were often below the quantification limit with ESI. For abundant sterols such as cholesterol, lanosterol, and desmosterol, both APCI and TPI provided comparable quantitative results, with small deviations attributed to matrix effects. Overall, APCI and TPI offered broader analyte coverage and greater robustness for sterol quantification in complex matrices compared to ESI, underlining their suitability for practical applications.

## Conclusion

A novel API source for LC-MS coupling based on TPI was developed and evaluated against established ion sources ESI and APCI for its suitability in sterol analysis. ESI exhibited substantial ion suppression, whereas APCI and TPI showed only minimal suppression, which is an important factor when transferring analytical methods to routine applications, as it directly affects quantification accuracy. In addition, signal stability was more consistent for APCI and TPI during an extended series of injections involving complex, high-matrix samples. Characterization showed that ESI is impractical to use and that APCI and TPI provided comparable LOD and LOQ values, although APCI often outperformed TPI. Application to human plasma, HepG2 cells, and liver tissue demonstrated the applicability of TPI for sterol quantification in challenging biological matrices, with results closely aligning with those obtained using APCI. This is notable given that this home-made TPI is still a prototype, yet already delivers performance almost comparable to established, commercially available ion sources.

## Supplementary Information

Below is the link to the electronic supplementary material.

Supplementary Material 1 (DOCX 330 KB)

## Data Availability

All data supporting the findings of the present study are included within the article and its Supplementary Information files or are available upon request from the authors.

## References

[1] Zhang H, Yang Y, Jiang Y, Zhang M, Xu Z, Wang X, Jiang J. Mass Spectrometry Analysis for Clinical Applications: A Review. Crit Rev Anal Chem; 2023. p. 1-20. 10.1080/10408347.2023.2274039 10.1080/10408347.2023.2274039 37910438

[2] Nasiri A, Jahani R, Mokhtari S, Yazdanpanah H, Daraei B, Faizi M, et al. Overview, consequences, and strategies for overcoming matrix effects in LC-MS analysis: a critical review. Analyst. 2021;146(20):6049–63. 10.1039/d1an01047f. 34546235 10.1039/d1an01047f

[3] Ayala-Cabrera JF, Montero L, Meckelmann SW, Uteschil F, Schmitz OJ. Review on atmospheric pressure ionization sources for gas chromatography-mass spectrometry. Part I: Current ion source developments and improvements in ionization strategies. Anal Chim Acta. 2023;1238:340353. 10.1016/j.aca.2022.340353. 36464440 10.1016/j.aca.2022.340353

[4] Fenn JB, Mann M, Meng CK, Wong SF, Whitehouse CM. Electrospray ionization for mass spectrometry of large biomolecules. Science. 1989;246(4926):64–71. 10.1126/science.2675315. 10.1126/science.2675315 2675315

[5] Alexandrov ML, Gall LN, Krasnov NV, Nikolaev VI, Pavlenko VA, Shkurov VA. Extraction of ions from solutions under atmospheric pressure as a method for mass spectrometric analysis of bioorganic compounds. Rapid Commun Mass Spectrom. 2008;22(3):267–70. 10.1002/rcm.3113. 10.1002/rcm.3113 18181250

[6] Banerjee S, Mazumdar S. Electrospray ionization mass spectrometry: a technique to access the information beyond the molecular weight of the analyte. Int J Anal Chem. 2012;2012:282574. 10.1155/2012/282574. 22611397 10.1155/2012/282574 PMC3348530

[7] Medina DAV, Maciel EVS, Lancas FM. Mass spectrometric detection, instrumentation, and ionization methods. Liquid Chromatogr. 2023;1:679–706. 10.1016/B978-0-323-99968-7.00016-3.

[8] Young LM, Saunders JC, Mahood RA, Revill CH, Foster RJ, Ashcroft AE, et al. ESI-IMS-MS: a method for rapid analysis of protein aggregation and its inhibition by small molecules. Methods. 2016;95:62–9. 10.1016/j.ymeth.2015.05.017. 26007606 10.1016/j.ymeth.2015.05.017 PMC4769093

[9] Totsch K, Fjeldsted JC, Stow SM, Schmitz OJ, Meckelmann SW. Effect of sampling rate and data pretreatment for targeted and nontargeted analysis by means of liquid chromatography coupled to drift time ion mobility quadruple time-of-flight mass spectrometry. J Am Soc Mass Spectrom. 2021;32(10):2592–603. 10.1021/jasms.1c00217. 34515480 10.1021/jasms.1c00217

[10] Kuchar L, Asfaw B, Rybova J, Ledvinova J. Tandem mass spectrometry of sphingolipids: applications for diagnosis of sphingolipidoses. Adv Clin Chem. 2016;77:177–219. 10.1016/bs.acc.2016.06.004. 27717417 10.1016/bs.acc.2016.06.004

[11] Shah S, Friedman SH. An ESI-MS method for characterization of native and modified oligonucleotides used for RNA interference and other biological applications. Nat Protoc. 2008;3(3):351–6. 10.1038/nprot.2007.535. 18323805 10.1038/nprot.2007.535

[12] Yang K, Han X. Accurate quantification of lipid species by electrospray ionization mass spectrometry - meet a key challenge in lipidomics. Metabolites. 2011;1(1):21–40. 10.3390/metabo1010021. 10.3390/metabo1010021 PMC3420347 22905337

[13] Chen L, Song F, Liu Z, Zheng Z, Xing J, Liu S. Study of the ESI and APCI interfaces for the UPLC-MS/MS analysis of pesticides in traditional Chinese herbal medicine. Anal Bioanal Chem. 2014;406(5):1481–91. 10.1007/s00216-013-7508-7. 10.1007/s00216-013-7508-7 24346143

[14] Carroll DI, Dzidic I, Horning EC, Stillwell RN. Atmospheric Pressure Ionization Mass Spectrometry. Appl Spectrosc Rev. 1981;17(3):337–406. 10.1002/9780470027318.a6003.

[15] Byrdwell WC. Atmospheric pressure chemical ionization mass spectrometry for analysis of lipids. Lipids. 2001;36(4):327–46. 10.1007/s11745-001-0725-5. 11383683 10.1007/s11745-001-0725-5

[16] Cardoso LV, Tomasini D, Sampaio MRF, Caldas SS, Kleemann N, Primel EG, et al. Optimization and validation of a method using SPE and LC-APCI-MS/MS for determination of pharmaceuticals in surface and public supply water. J Braz Chem Soc. 2011;22(10):1944–52. 10.1590/s0103-50532011001000016.

[17] Zarrouk W, Carrasco-Pancorbo A, Zarrouk M, Segura-Carretero A, Fernandez-Gutierrez A. Multi-component analysis (sterols, tocopherols and triterpenic dialcohols) of the unsaponifiable fraction of vegetable oils by liquid chromatography-atmospheric pressure chemical ionization-ion trap mass spectrometry. Talanta. 2009;80(2):924–34. 10.1016/j.talanta.2009.08.022. 19836574 10.1016/j.talanta.2009.08.022

[18] Carretero AS, Carrasco-Pancorbo A, Cortacero S, Gori A, Cerretani L, Fernández-Gutiérrez A. A simplified method for HPLC-MS analysis of sterols in vegetable oil. Eur J Lipid Sci Technol. 2008;110(12):1142–9. 10.1002/ejlt.200700237.

[19] Haraguchi K, Kato Y, Atobe K, Okada S, Endo T, Matsubara F, et al. Negative APCI-LC/MS/MS method for determination of natural persistent halogenated products in marine biota. Anal Chem. 2008;80(24):9748–55. 10.1021/ac801824f. 19012416 10.1021/ac801824f

[20] Gors PE, Wittenhofer P, Ayala-Cabrera JF, Meckelmann SW. Potential of atmospheric pressure ionization sources for the analysis of free fatty acids in clinical and biological samples by gas chromatography-mass spectrometry. Anal Bioanal Chem. 2022;414(22):6621–34. 10.1007/s00216-022-04223-z. 35851410 10.1007/s00216-022-04223-z PMC9411222

[21] Martínez-Jarquín S, Winkler R. Low-temperature plasma (LTP) jets for mass spectrometry (MS): ion processes, instrumental set-ups, and application examples. TrAC Trends Anal Chem. 2017;89:133–45. 10.1016/j.trac.2017.01.013.

[22] Cody RB, Laramee JA, Durst HD. Versatile new ion source for the analysis of materials in open air under ambient conditions. Anal Chem. 2005;77(8):2297–302. 10.1021/ac050162j. 10.1021/ac050162j 15828760

[23] Snyder DT, Pulliam CJ, Ouyang Z, Cooks RG. Miniature and fieldable mass spectrometers: recent advances. Anal Chem. 2016;88(1):2–29. 10.1021/acs.analchem.5b03070. 26422665 10.1021/acs.analchem.5b03070 PMC5364034

[24] Mirabelli MF, Gionfriddo E, Pawliszyn J, Zenobi R. Fast screening of illicit drugs in beverages and biological fluids by direct coupling of thin film microextraction to dielectric barrier discharge ionization-mass spectrometry. Analyst. 2019;144(8):2788–96. 10.1039/c8an02448k. 10.1039/c8an02448k 30869658

[25] Knodel A, Foest D, Brandt S, Ahlmann N, Marggraf U, Gilbert-Lopez B, et al. Detection and evaluation of lipid classes and other hydrophobic compounds using a laser desorption/plasma ionization interface. Anal Chem. 2020;92(22):15212–20. 10.1021/acs.analchem.0c03839. 33135875 10.1021/acs.analchem.0c03839

[26] Gilbert-Lopez B, Schilling M, Ahlmann N, Michels A, Hayen H, Molina-Diaz A, et al. Ambient diode laser desorption dielectric barrier discharge ionization mass spectrometry of nonvolatile chemicals. Anal Chem. 2013;85(6):3174–82. 10.1021/ac303452w. 23419061 10.1021/ac303452w

[27] Ayala-Cabrera JF, Turkowski J, Uteschil F, Schmitz OJ. Development of a tube plasma ion source for gas chromatography-mass spectrometry analysis and comparison with other atmospheric pressure ionization techniques. Anal Chem. 2022;94(27):9595–602. 10.1021/acs.analchem.2c00582. 10.1021/acs.analchem.2c00582 35758294

[28] Vogel P, Lazarou C, Gazeli O, Brandt S, Franzke J, Moreno-Gonzalez D. Study of controlled atmosphere flexible microtube plasma soft ionization mass spectrometry for detection of volatile organic compounds as potential biomarkers in saliva for cancer. Anal Chem. 2020;92(14):9722–9. 10.1021/acs.analchem.0c01063. 32579344 10.1021/acs.analchem.0c01063

[29] Pape A, Schmitz OJ. Dielectric barrier discharge in mass spectrometry – An overview over plasma investigations and ion sources applications*.* TrAC Trends Anal Chem. 2024;170. 10.1016/j.trac.2023.117420

[30] Hayen H, Michels A, Franzke J. Dielectric barrier discharge ionization for liquid chromatography/mass spectrometry. Anal Chem. 2009;81(24):10239–45. 10.1021/ac902176k. 10.1021/ac902176k 19911793

[31] Horvatic V, Vadla C, Franzke J. Discussion of fundamental processes in dielectric barrier discharges used for soft ionization. Spectrochim Acta B At Spectrosc. 2014;100:52–61. 10.1016/j.sab.2014.08.010.

[32] Aldea E, Peeters P, De Vries H, Van De Sanden MCM. Atmospheric glow stabilization. Do we need pre-ionization? Surf Coat Technol. 2005;200(1–4):46–50. 10.1016/j.surfcoat.2005.01.052.

[33] Hong Y, Yoo S, Lee B. An atmospheric-pressure nitrogen-plasma jet produced from microdischarges in a porous dielectric. J Electrostat. 2011;69(2):92–6. 10.1016/j.elstat.2011.01.002.

[34] Hong YC, Uhm HS, Yi WJ. Atmospheric pressure nitrogen plasma jet: observation of striated multilayer discharge patterns. Appl Phys Lett. 2008. 10.1063/1.2969287.

[35] Babaeva NY, Naidis GV, Panov VA, Wang R, Zhao Y, Shao T. Interaction of argon and helium plasma jets and jets arrays with account for gravity. Phys Plasmas. 2018. 10.1063/1.5024778.

[36] Höft H, Kettlitz M, Becker MM, Hoder T, Loffhagen D, Brandenburg R, et al. Breakdown characteristics in pulsed-driven dielectric barrier discharges: influence of the pre-breakdown phase due to volume memory effects. J Phys D Appl Phys. 2014. 10.1088/0022-3727/47/46/465206.

[37] Jiang PC, Wang WC, Zhang S, Jia L, Yang DZ, Tang K, et al. An uniform DBD plasma excited by bipolar nanosecond pulse using wire-cylinder electrode configuration in atmospheric air. Spectrochim Acta A Mol Biomol Spectrosc. 2014;122:107–12. 10.1016/j.saa.2013.10.004. 24299982 10.1016/j.saa.2013.10.004

[38] Harper JD, Charipar NA, Mulligan CC, Zhang X, Cooks RG, Ouyang Z. Low-temperature plasma probe for ambient desorption ionization. Anal Chem. 2008;80(23):9097–104. 10.1021/ac801641a. 10.1021/ac801641a 19551980

[39] Brecht D, Uteschil F, Schmitz OJ. Development of an inverse low-temperature plasma ionization source for liquid chromatography/mass spectrometry. Rapid Commun Mass Spectrom. 2021;35(10):e9071. 10.1002/rcm.9071. 33625792 10.1002/rcm.9071

[40] Gilbert-Lopez B, Geltenpoth H, Meyer C, Michels A, Hayen H, Molina-Diaz A, et al. Performance of dielectric barrier discharge ionization mass spectrometry for pesticide testing: a comparison with atmospheric pressure chemical ionization and electrospray ionization. Rapid Commun Mass Spectrom. 2013;27(3):419–29. 10.1002/rcm.6469. 23280973 10.1002/rcm.6469

[41] Lara-Ortega FJ, Robles-Molina J, Brandt S, Schutz A, Gilbert-Lopez B, Molina-Diaz A, et al. Use of dielectric barrier discharge ionization to minimize matrix effects and expand coverage in pesticide residue analysis by liquid chromatography-mass spectrometry. Anal Chim Acta. 2018;1020:76–85. 10.1016/j.aca.2018.02.077. 10.1016/j.aca.2018.02.077 29655430

[42] Harris GA, Galhena AS, Fernandez FM. Ambient sampling/ionization mass spectrometry: applications and current trends. Anal Chem. 2011;83(12):4508–38. 10.1021/ac200918u. 21495690 10.1021/ac200918u

[43] Roach PJ, Laskin J, Laskin A. Nanospray desorption electrospray ionization: an ambient method for liquid-extraction surface sampling in mass spectrometry. Analyst. 2010;135(9):2233–6. 10.1039/c0an00312c. 20593081 10.1039/c0an00312c

[44] Lobbecke S, Pape A, Montero L, Uteschil F, Ayala-Cabrera JF, Schmitz OJ. Improving the reliability of phthalate esters analysis in water samples by gas chromatography-tube plasma ionization-high-resolution mass spectrometry (GC-TPI-HRMS). Talanta. 2025;285:127388. 10.1016/j.talanta.2024.127388. 39700716 10.1016/j.talanta.2024.127388

[45] Mullen PJ, Yu R, Longo J, Archer MC, Penn LZ. The interplay between cell signalling and the mevalonate pathway in cancer. Nat Rev Cancer. 2016;16(11):718–31. 10.1038/nrc.2016.76. 10.1038/nrc.2016.76 27562463

[46] King RJ, Singh PK, Mehla K. The cholesterol pathway: impact on immunity and cancer. Trends Immunol. 2022;43(1):78–92. 10.1016/j.it.2021.11.007. 10.1016/j.it.2021.11.007 PMC8812650 34942082

[47] Pelton K, Freeman MR, Solomon KR. Cholesterol and prostate cancer. Curr Opin Pharmacol. 2012;12(6):751–9. 10.1016/j.coph.2012.07.006. 22824430 10.1016/j.coph.2012.07.006 PMC3515742

[48] Zhao X, Lian X, Xie J, Liu G. Accumulated cholesterol protects tumours from elevated lipid peroxidation in the microenvironment. Redox Biol. 2023;62:102678. 10.1016/j.redox.2023.102678. 10.1016/j.redox.2023.102678 PMC10036943 36940607

[49] Gosetti F, Mazzucco E, Zampieri D, Gennaro MC. Signal suppression/enhancement in high-performance liquid chromatography tandem mass spectrometry. J Chromatogr A. 2010;1217(25):3929–37. 10.1016/j.chroma.2009.11.060. 20004403 10.1016/j.chroma.2009.11.060

[50] European Medicines Agency and International Council for Harmonisation of Technical Requirements for Pharmaceuticals for Human Use. ICH Q2(R2) Guideline on validation of analytical procedures. 2024.

[51] Wittenhofer P, Kiesewetter L, Schmitz OJ, Meckelmann SW. Investigation of the cholesterol biosynthesis by heart-cut liquid chromatography and mass spectrometric detection. J Chromatogr A. 2024;1738:465475. 10.1016/j.chroma.2024.465475. 39488880 10.1016/j.chroma.2024.465475

[52] King R, Bonfiglio R, Fernandez-Metzler C, Miller-Stein C, Olah T. Mechanistic investigation of ionization suppression in electrospray ionization. J Am Soc Mass Spectrom. 2000;11(11):942–50. 10.1016/S1044-0305(00)00163-X. 11073257 10.1016/S1044-0305(00)00163-X

[53] Jaeger C, Lisec J. Towards Unbiased Evaluation of Ionization Performance in LC-HRMS Metabolomics Method Development. Metabolites. 2022;**12**(5). 10.3390/metabo12050426 10.3390/metabo12050426 PMC9144264 35629930

[54] Simon-Manso Y, Lowenthal MS, Kilpatrick LE, Sampson ML, Telu KH, Rudnick PA, et al. Metabolite profiling of a NIST standard reference material for human plasma (SRM 1950): GC-MS, LC-MS, NMR, and clinical laboratory analyses, libraries, and web-based resources. Anal Chem. 2013;85(24):11725–31. 10.1021/ac402503m. 24147600 10.1021/ac402503m

