# Supplementary material for: Sterol analysis in cancer cells using atmospheric pressure ionization techniques
Source: Anal Bioanal Chem. 2025 Oct 2;417(28):6379–90. doi: 10.1007/s00216-025-06126-1 (PMC12596357; doi:10.1007/s00216-025-06126-1)
Supplement: Supplementary file 1 — Supplementary Material 1 (DOCX 330 KB) [file 216_2025_6126_MOESM1_ESM.docx]

**Supplementary Material for:**

**Sterol Analysis in Cancer Cells Using Atmospheric Pressure Ionization Techniques**

Pia Wittenhofer^1^, Juan F. Ayala-Cabrera^1,2^, Laila Orell^1^, Florian Uteschil^1^, Sven W. Meckelmann^1^*, Oliver J. Schmitz^1^*

^1^Applied Analytical Chemistry, University of Duisburg-Essen, Universitaetsstrasse 5, 45141 Essen, Germany

^2^Department of Analytical Chemistry, University of the Basque Country (UPV/EHU),
Sarriena Auzoa, 48940 Leioa, Spain

Corresponding authors:

*Sven W. Meckelmann

Applied Analytical Chemistry, University of Duisburg-Essen, Universitaetsstrasse 5, 45141 Essen, Germany https://orcid.org/0000-0002-0407-7879 E-mail: sven.meckelmann@uni-due.de

*Oliver J. Schmitz

Applied Analytical Chemistry, University of Duisburg-Essen, Universitaetsstrasse 5, 45141 Essen, Germany https://orcid.org/0000-0002-0407-7879 E-mail: oliver.schmitz@uni-due.de

***Sample preparation***

For the extraction of free and esterified sterols, 10 µL human plasma, 1 mio cells or 10 mg tissue were mixed with 10 µL of internal standard mix (10 µmol/L lathosterol ^2^H_7_, lanosterol ^2^H_6_, desmosterol ^2^H_6_, T-MAS ^2^H_6_, zymosterol ^2^H_5_, dihydrolanosterol ^2^H_7,_  100 µmol/L of cholesterol ^2^H_7_ in methanol) and 100 µL methanol. The samples were hydrolyzed using 60 µL 10 mol/L potassium hydroxide solution. The samples were vortexed for 1 min, homogenized in an ultrasonic bath for 5 min and incubated at 60 °C for one hour. Afterwards, 70 µL 50 % acetic acid was added and sterols were extracted by the addition of 200 µL water, 500 µL of acetic acid/propan-2-ol/n-hexane (2:20:30, v/v/v). The samples were vortexed for 1 min and phase separation was induced by the addition of 500 µL n-hexane. After vortexing for 1 min the samples were centrifugated at 2000 g for 2 min and the organic phase was collected into a new vial. The remaining phase was extracted with 400 µL n-hexane and 100 µL water. After vortexing for 1 min and centrifugation at 2000 g for 2 min the organic phase was collected. The remaining phase was washed with 500 µL n-hexane, vortexed for 1 min and centrifugated at 2000 g for 2 min. The organic phase was collected, and the combined phases were dried at 45 °C in a vacuum evaporator, resuspend in 100 µL methanol and directly analyzed or stored at -80 °C until analysis.

# ***Figures***


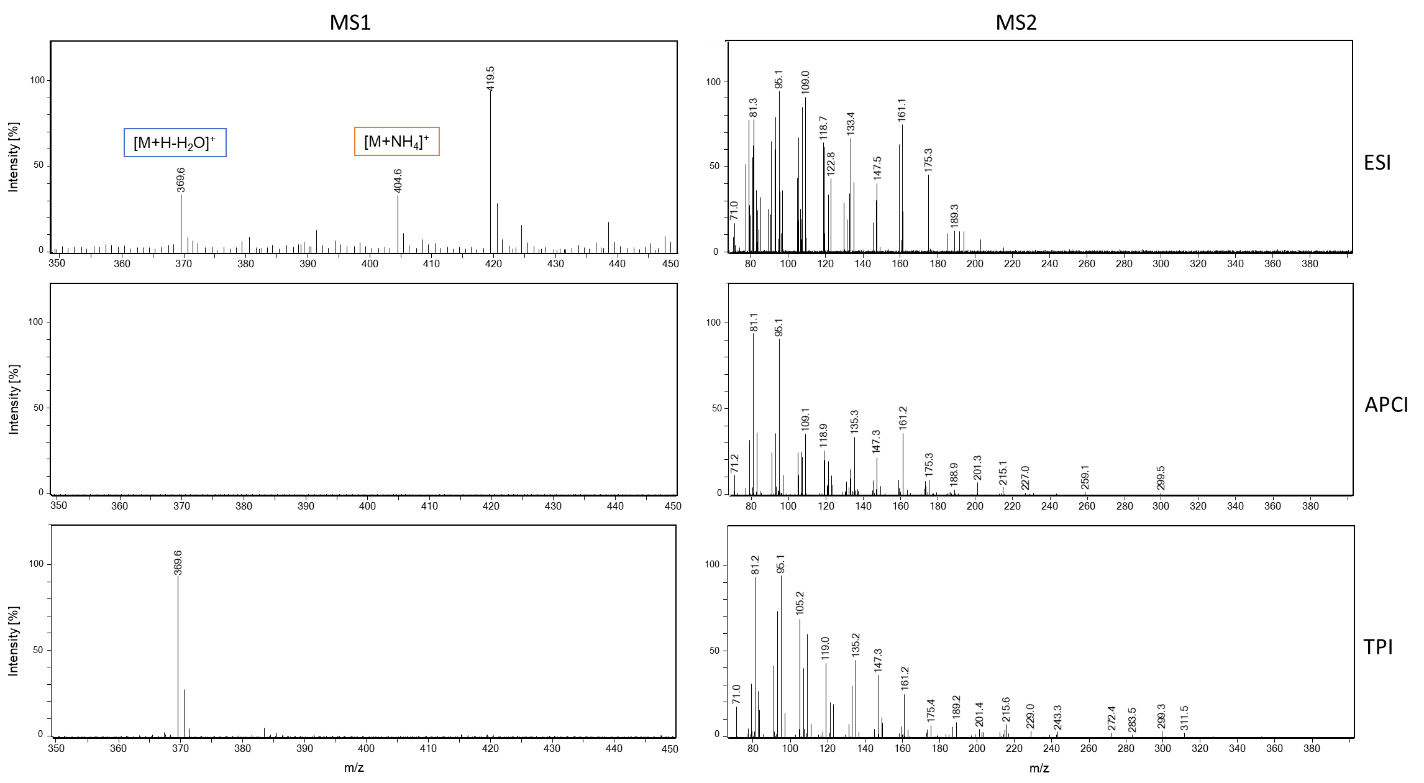


**Figure S1**: Adduct formation (MS1, left) and fragmentation pattern (MS2, right) of cholesterol using ESI, APCI, and TPI ionization techniques.


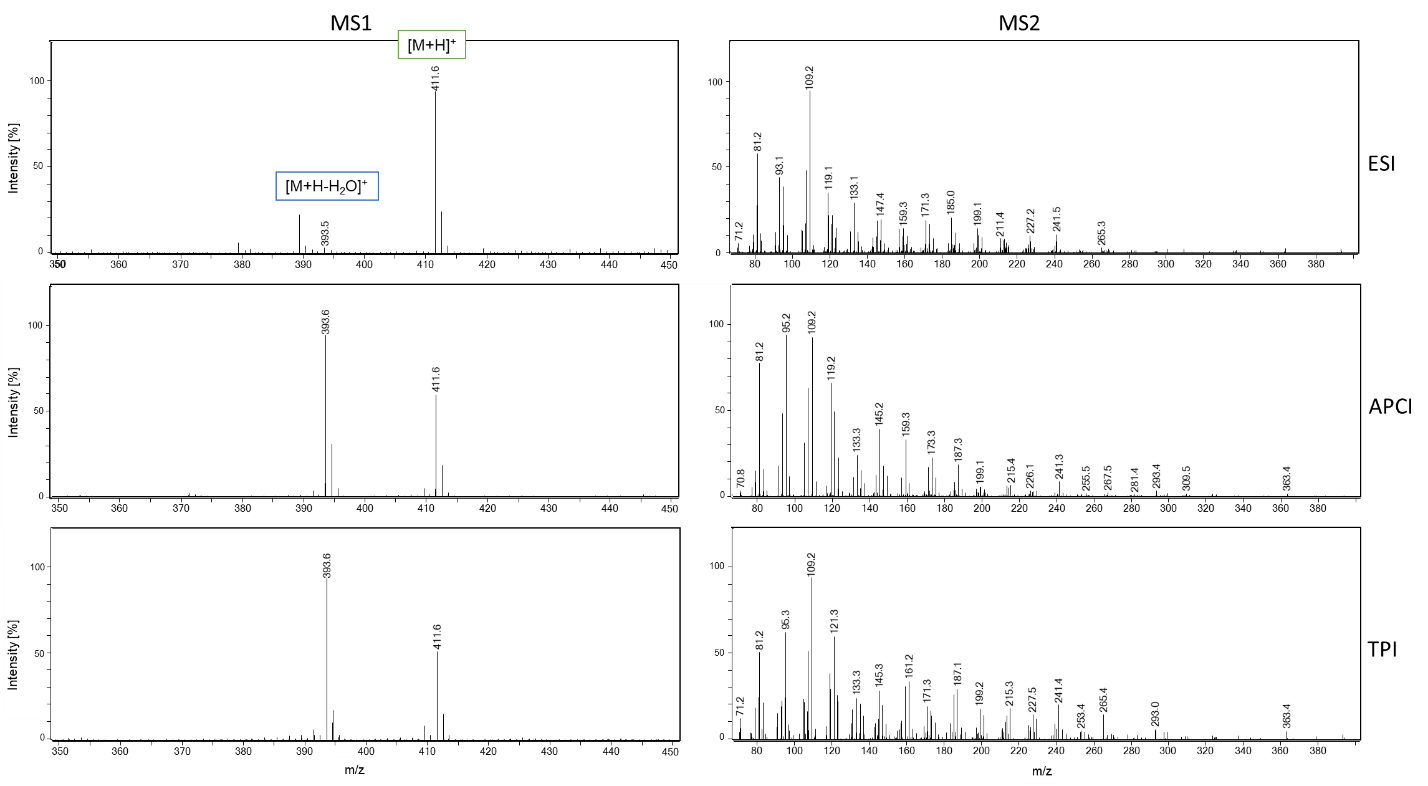


**Figure S2**: Adduct formation (MS1, left) and fragmentation pattern (MS2, right) of FF-MAS using ESI, APCI, and TPI ionization techniques.

**Figure S3**: Contour plots of TPI source conditions for (a) lanosterol, (b) FFMAS, (c) dihydro T-MAS and (d) cholesterol.

**Figure S4**: Signal stability of ESI, APCI, and TPI ion sources evaluated using human plasma (left) and liver tissue (right) samples over 26 hours and a total of 40 consecutive injections. Analysis was performed using 2D-LC, and the cholesterol peak area was considered for evaluation.

**Figure S5:** Box plots of the LOQ values of the investigated biosynthesis related compounds using ESI, APCI, and TPI as ion sources.

**Figure S6**: Analysis of sterols extracted from human plasma. The same extracts were analyzed and quantified using heart-cut 2D LC and QqQ MS with ESI, APCI or TPI as ion source (n = 3).

**Figure S7**: Analysis of extracted liver tissue using n-hexane. The same extracts were analyzed and quantified using heart-cut 2D LC and QqQ MS with ESI, APCI or TPI as ion source (n = 3).

***Tables***

**Table S1**: Transition and source parameter of sMRM of cholesterol biosynthesis for TPI, ESI and APCI.

|  |  |  |  |  | Collision energy [V] | | | Cell accelerator voltage [V] | | |
| --- | --- | --- | --- | --- | --- | --- | --- | --- | --- | --- |
| Compound | Precursor ion *m/z* | Product ion *m/z* | Retention time* [min] | Delta retention time for QqQ transition* [min] | ESI | APCI | TPI | ESI | APCI | TPI |
| 2,3-Oxidosqualene | 427.4 | 95.0 | 9.18 | 4 | 30 | 30 | 40 | 1 | 1 | 3 |
| 7-Dehydrocholesterol | 367.3 | 95.0 | 7.70 | 4 | 40 | 40 | 30 | 2 | 3 | 1 |
| Cholesterol | 369.4 | 95.0 | 8.45 (22.1) | 4 (8) | 40^!1^ (0^!2^) | 35^!1^ (0^!2^) | 40^!1^ (0^!2^) | 3 | 4 | 2 |
| Cholesterol (^2^H_7_) | 376.4 | 95.0 | 8.45 (22.1) | 4 (8) | 40 | 35 | 40 | 3 | 4 | 2 |
| Dehydrodesmosterol | 365.3 | 95.0 | 6.03 | 4 | 30 | 35 | 45 | 1 | 1 | 1 |
| Dehydrolathosterol | 367.3 | 95.0 | 5.98 | 4 | 40 | 45 | 45 | 2 | 2 | 3 |
| Desmosterol | 367.3 | 95.0 | 6.57 | 4 | 45 | 40 | 45 | 4 | 4 | 2 |
| Desmosterol (^2^H_6_) | 373.3 | 95.0 | 6.57 | 4 | 45 | 40 | 45 | 4 | 4 | 2 |
| Dihydro-FF-MAS | 395.4 | 95.0 | 9.90 | 4 | 40 | 40 | 45 | 1 | 4 | 2 |
| Dihydrolanosterol | 411.4 | 95.0 | 11.63 | 4 | 45 | 45 | 40 | 1 | 1 | 1 |
| Dihydrolanosterol (^2^H_7_) | 418.4 | 95.0 | 11.63 | 4 | 45 | 45 | 40 | 1 | 1 | 1 |
| Dihydro-T-MAS | 397.4 | 95.0 | 10.85 | 4 | 40 | 40 | 40 | 1 | 5 | 1 |
| FF-MAS | 411.4 | 95.0 | 7.73 | 4 | 45 | 40 | 45 | 2 | 2 | 1 |
| Lanosterol | 409.4 | 95.0 | 13.66 | 4 | 45 | 40 | 45 | 1 | 3 | 1 |
| Lanosterol (^2^H_6_) | 415.4 | 95.0 | 13.66 | 4 | 45 | 40 | 45 | 1 | 3 | 1 |
| Lathosterol | 369.4 | 95.0 | 7.66 | 4 | 40 | 40 | 45 | 2 | 3 | 3 |
| Lathosterol (^2^H_7_) | 376.4 | 95.0 | 7.66 | 4 | 40 | 40 | 45 | 2 | 3 | 3 |
| Squalene | 411.4 | 95.0 | 15.55 | 4 | 40 | 40 | 35 | 2 | 5 | 3 |
| T-MAS | 395.4 | 95.0 | 8.52 (22.3) | 4 (8) | 40 | 45 | 40 | 1 | 1 | 3 |
| T-MAS (^2^H_6_) | 401.4 | 95.0 | 8.52 (22.3) | 4 (8) | 40 | 45 | 40 | 1 | 1 | 3 |
| Zymostenol | 369.4 | 95.0 | 7.26 | 4 | 40 | 40 | 40 | 2 | 1 | 2 |
| Zymosterol | 367.3 | 95.0 | 5.69 | 4 | 45 | 40 | 40 | 3 | 4 | 3 |
| Zymosterol (^2^H_5_) | 372.3 | 95.0 | 5.69 | 4 | 45 | 40 | 40 | 3 | 4 | 3 |

*Values for heart-cut 2D-LC-MS in brackets !1: for low concentration, !2: for high concentration

**Table S2**: Method characterization (1D-LC-QqQ-MS) according to ICH Guideline [1] .

| Analyte | Internal standard | LOD [nmol/L]^(a)^ | | | Linear range [nmol/L] | | | | | | slope | | | R^2 (c)^ | | |
| --- | --- | --- | --- | --- | --- | --- | --- | --- | --- | --- | --- | --- | --- | --- | --- | --- |
|  |  |  |  |  | LOQ^(b)^ | | | Upper LOQ | | |  |  |  |  |  |  |
|  |  | ESI | APCI | TPI | ESI | APCI | TPI | ESI | APCI | TPI | ESI | APCI | TPI | ESI | APCI | TPI |
| 2,3-Oxidosqualene | Lanosterol-^2^H_6_ | 1,000 | 300 | 1,000 | 3,000 | 300 | 1,000 | 30,000 | 60,000 | 60,000 | 2.33 | 3.92 | 4.16 | 0.91 | 0.92 | 0.97 |
| 7-Dehydrocholesterol | Cholesterol-^2^H_7_ | 100 | 10 | 100 | 300 | 30 | 100 | 10,000 | 60,000 | 60,000 | 2.29 | 0.94 | 0.90 | 0.97 | 0.97 | 0.96 |
| Cholesterol | Cholesterol-^2^H_7_ | 300 | 3 | 30 | 300 | 10 | 100 | 10,000 | 60,000 | 60,000 | 3.59 | 2.99 | 2.85 | 0.95 | 0.95 | 0.87 |
| Dehydrodesmosterol | Desmosterol-^2^H_6_ | 300 | 30 | 30 | 1,000 | 30 | 30 | 90,000 | 60,000 | 90,000 | 2.47 | 2.62 | 3.09 | 0.90 | 0.97 | 0.96 |
| Dehydrolathosterol | Zymosterol-^2^H_5_ | 1,000 | 10 | 30 | 1,000 | 30 | 30 | 10,000 | 60,000 | 60,000 | 1.46 | 2.12 | 2.39 | 0.89 | 0.94 | 0.97 |
| Desmosterol | Desmosterol-^2^H_6_ | 300 | 10 | 30 | 1,000 | 30 | 30 | 90,000 | 60,000 | 60,000 | 2.01 | 2.36 | 2.31 | 0.99 | 0.97 | 0.91 |
| Dihydro-FF-MAS | T-MAS-^2^H_6_ | 100 | 10 | 10 | 300 | 30 | 30 | 30,000 | 60,000 | 90,000 | 2.45 | 1.58 | 2.27 | 0.95 | 0.97 | 0.98 |
| Dihydrolanosterol | Dihydrolanosterol-^2^H_7_ | 1,000 | 10 | 30 | 1,000 | 10 | 30 | 60,000 | 60,000 | 90,000 | 1.05 | 1.05 | 1.03 | 0.98 | 0.98 | 0.98 |
| Dihydro-T-MAS | T-MAS-^2^H_6_ | 100 | 3 | 10 | 300 | 10 | 30 | 30,000 | 60,000 | 90,000 | 1.61 | 3.03 | 2.91 | 0.97 | 0.97 | 0.97 |
| FF-MAS | T-MAS-^2^H_6_ | 300 | 3 | 30 | 300 | 10 | 30 | 30,000 | 90,000 | 90,000 | 5.57 | 1.01 | 1.23 | 0.98 | 0.94 | 0.95 |
| Lanosterol | Lanosterol-^2^H_6_ | 1,000 | 3 | 30 | 1,000 | 10 | 100 | 10,000 | 60,000 | 30,000 | 0.71 | 1.48 | 1.95 | 0.97 | 0.96 | 0.99 |
| Lathosterol | Lathosterol-^2^H_7_ | 1,000 | 30 | 100 | 1,000 | 30 | 100 | 10,000 | 60,000 | 60,000 | 1.30 | 1.22 | 1.21 | 0.95 | 0.96 | 0.93 |
| Squalene | Lanosterol-^2^H_6_ | 10,000 | 1,000 | 1,000 | 10,000 | 1,000 | 1,000 | 90,000 | 60,000 | 30,000 | 0.10 | 0.45 | 0.39 | 0.74 | 0.97 | 0.99 |
| T-MAS | T-MAS-^2^H_6_ | 100 | 10 | 10 | 100 | 10 | 30 | 10,000 | 90,000 | 90,000 | 2.53 | 2.44 | 2.65 | 0.98 | 0.97 | 0.96 |
| Zymostenol | Zymosterol-^2^H_5_ | 1,000 | 30 | 100 | 1,000 | 30 | 100 | 30,000 | 60,000 | 60,000 | 2.68 | 4.23 | 4.54 | 0.89 | 0.97 | 0.95 |
| Zymosterol | Zymosterol-^2^H_5_ | 300 | 3 | 30 | 1,000 | 10 | 30 | 10,000 | 60,000 | 60,000 | 2.33 | 3.92 | 4.16 | 0.85 | 0.95 | 0.97 |
| Median value | | 1,000 | 10 | 30 | 1,000 | 10 | 30 | 10,000 | 60,000 | 60,000 | 2.40 | 2.43 | 2.62 | 0.93 | 0.96 | 0.96 |

*at LOQ

^(a)^ S/N ≥ 3

^(b)^ S/N ≥ 9 and accuracy ± 20%

^(c)^ R^2^ of a 1/x^2^ weighted calibration curve

**References**

1. European Medicin Agency and International Council for Harmonisation of Technical Requirements for Pharmaceuticals for Human Use. ICH Q2(R2) Guideline on validation of analytical procedures 2024
